# Laser-Induced Damage of UHMW-PE-Based Layered Ballistic Materials

**DOI:** 10.1021/acsomega.4c04642

**Published:** 2024-11-08

**Authors:** Can Candan, Emine Yasemen Kaya Çekin, Elif Türkan Akşit Kaya, Mehmet Tiken, Alpan Bek, Halil Berberoğlu, Elif Orhan, Aydın Yeniay

**Affiliations:** † Department of Physics, Middle East Technical University, 06800 Ankara, Turkey; ‡ TUBITAK-BILGEM, Electro-Optics and Laser Systems Group, 41470 Gebze, Turkey; § Department of Physics, Faculty of Science, Gazi University, 06560 Ankara, Turkey; ∥ Polatlı Faculty of Science and Letters, Physics Department, Ankara Hacı Bayram Veli University, 06900 Ankara, Turkey

## Abstract

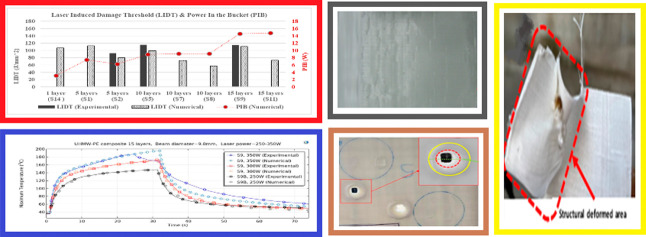

The laser-induced damage threshold properties of material structures play a key role in identifying and selecting optimum materials with the respective geometric configurations for laser shielding applications. The laser-induced damage mechanism is predominantly influenced by the absorption, thermal conductivity, and transition temperature characteristics of the materials. Ultrahigh-molecular weight polyethylene (UHMW-PE) ballistic composite sheet structures, traditionally employed for conventional ballistic purposes, merit examination for their laser shielding capabilities, leveraging their established use in conventional shielding applications. These materials can be configured into the desired geometries along with layer structures. In this study, we conducted both numerical modeling and experimental investigations to assess and analyze the laser-induced damage mechanism in layered UHMW-PE material structures. To the best of our knowledge, this is the first time that both numerical modeling and experimental investigations have been conducted to assess and analyze the laser-induced damage mechanisms in such layered UHMW-PE material structures for high-power laser exposure. Our laser-material interaction model takes into account the optical, thermal, and structural properties of such layered UHMW-PE composite materials. Our model-based numerical calculations consistently align with experimental results, providing validated analysis of the laser-induced damage mechanism concerning layered structure geometries, optical power, and density (i.e., up to 5 kW at 1075 nm wavelength), as well as revealing nonlinear behavior in certain cases

## 1 Introduction

With the advent of high-power single-mode fiber lasers, the development of laser-based directed energy systems with optical power levels reaching kilowatt levels has become feasible for operational use across various fields.^1,2^ Ensuring laser safety and implementing effective shielding against laser exposure have become imperative to prevent health hazards beyond controlled laboratory environments. Among the main requirements for laser shielding materials are high laser-induced damage threshold (LIDT) levels, lightweight, small size, and low cost for practical applications in the fields. Besides laser shielding aspects, laser-induced damage in optical components is also a concern in laser-based directed energy optical systems where laser-induced phase distortions give rise to degradation of system performance and, in turn, may even lead to catastrophic system failure.^3^ In analyzing laser-induced damage mechanisms, optical, thermal, and structural properties of materials eventually affect respective LIDT levels and system performance for given laser parameters (i.e., power, wavelength, beam diameter, duration, etc.).^4^ According to ISO 21254 standards, LIDT is defined as the “highest quantity of laser radiation incident upon the optical component for which the extrapolated probability of damage is zero”. Generally, the purpose of LIDT is to specify the maximum laser intensity for continuous wave (CW) lasers (W/cm^2^) that a laser optic can withstand before permanent damage occurs.^5^ On the other hand, for shielding applications, LIDT levels can be considered the upper limit of laser intensity along with the duration that shielding material can prevent the laser from passing through. In addition, in laser-based directed energy applications, there is another criterion called “all-purpose damage criteria”, which is defined as the energy required to vaporize a cubic centimeter of most materials, i.e., 10^4^ J. Most directed energy systems damage targets when they are capable of delivering a fluency of about 10^4^ J/cm^2^.^6^

Regarding laser properties, power, wavelength, and beam product parameters are key factors in the laser-induced damage process. In LIDT and all-damage criteria analysis, power density (i.e., the optical power per unit area, typically measured in W/cm^2^) is mainly considered for CW lasers. However, in some cases, the power density may become extremely large with the reduction of the beam size (e.g., GW/cm^2^ fluency level for a 1 W laser with a 10 μm spot size), yet laser-induced effects may still be limited by the lack of total power. Therefore, another metric indicating the total power within an area, i.e., power in the bucket (PIB), is to be considered for high-power laser systems and materials processing applications. PIB describes how much laser power is integrated over a specified “bucket”, most often a spot of a specific radius at the material surface being illuminated. In addition, the intensity profile of the laser is simply the distribution of the intensity across a cross-section of the beam. Some of the most common intensity profiles are flat-top beams and Gaussian beams. Flat-top beams, or top-hat beams, have an intensity profile that is constant across the cross-section of the beam. Gaussian beams have an intensity profile that decreases as the distance from the center of the beam increases, following a Gaussian function. Single-mode lasers typically yield fundamental mode (i.e., HE11 for single-mode fibers) with an ideal Gaussian profile where the peak fluency is twice as large as that of a flat-top beam with the same optical power. Moreover, laser damage testing typically reveals a statistical nature that is dependent on several factors such as laser power, beam diameter, the number of test areas per sample, and the number of samples tested. Then, the fluency below the damage probability is specified as the damage threshold level of the respective sample.

The laser-induced damage mechanism is predominantly influenced by the absorption, thermal conductivity (TC), and transition temperature characteristics of materials. CW infrared lasers are known for their ability to induce material damage through interaction, a phenomenon primarily dependent on heat transfer dynamics.^7^ The heat conveyed onto a material surface by a laser is intricately linked to its optical absorption coefficient. This coefficient depends on various laser attributes such as intensity, wavelength, polarization, angle of incidence, and material-specific factors like composition, temperature, surface texture, oxide layer presence, and contamination level. The literature typically provides absorption coefficient data derived from ideal uncontaminated samples,^4^ requiring laboratory-based measurements for determining the more accurate absorption coefficient of a specific target sample. Commonly employed methods include laser calorimetry for direct assessment and reflectometry for indirect evaluation. Laser-induced energy absorption results in a temperature increase at the material surface, the extent of which depends on the absorbed energy. Precise knowledge of the sample’s emissivity value is essential for temperature measurement through noncontact methods based on blackbody radiation. Emissivity, representing the ratio of radiation emitted by objects at specific temperatures and wavelengths to that of blackbodies under identical conditions, follows Kirchhoff’s thermal radiation law.

The primary purpose of laser shielding is to prevent laser energy from passing through the shielding material. Laser shielding methods are commonly based on either reflection or absorption.^8^ The reflection mechanism, which aims to dissipate laser energy via highly reflective surfaces (e.g., high reflective coatings) at the laser wavelength, is an effective shielding method.^9^ However, redirecting laser energy still presents a risk of laser exposure to nearby areas. Additionally, using reflective surfaces is not practical in operational fields, as they increase overall visibility. On the other hand, absorption-based shielding can dissipate laser energy through ablation (e.g., thermochemical reactions) or thermal conduction (e.g., laser-induced heat energy transfer to the substrate via thermal conduction).^10^ The ablation mechanism often changes the shape of the material, which is especially unfavorable for targets with aerodynamic features, such as aircraft, UAVs, and drones.

Ideal laser shielding materials should demonstrate relatively high absorption at the laser wavelength and thermal durability with either high TC or high transition temperature to achieve high LIDT levels. Additionally, the lightweight, compact size, and low-cost features are important for practical applications outside of the laboratory environment. Regarding TC, metals such as copper or aluminum alloys are commonly utilized as heat-dissipation materials for many applications.^6^ Traditional thermal management materials, including metals and ceramics, exhibit high TC; however, their applicability is limited due to poor processability, high density, and high cost.^11,12^

On the other hand, polymers offer superior mechanical properties, chemical resistance, lightweight, electrical insulation, and ease of processing, which is why polymer-based thermal management materials have aroused great interest among researchers.^13^ The TC of bulk polymers is very low (<0.5 W/mK) since the polymer chains in bulk polymers are randomly oriented with a significant number of crystal–amorphous interfaces, defects, chain ends, and voids. These factors together contribute to strong phonon scattering, resulting in extremely low TC.^14^ The difference of TC between single-chain and bulk polymers is caused by the value of the phonon mean free path. However, Henry and Chen^15^ found that the TC of polyethylene (PE) chains can be as high as 350 W/mK by using molecular dynamics simulations. Ultrahigh-molecular weight polyethylene (UHMW-PE) is a type of PE that has extremely long polymer chains, resulting in a high molecular weight. UHMW-PE ballistic composite sheet structures traditionally employed with a proven track record of efficiency for conventional ballistic purposes also exhibit promising features against laser-induced damage. UHMW-PE, a linear homopolymer with (CH_2_–CH_2_–ethylene) *n* as the repeat unit, forms chains that align and compact into larger, well-oriented crystalline domains. UHMW-PE is a semicrystalline polymer, which contains crystalline and amorphous regions. These domains boast an average molecular weight between 3,500,000 and 7,500,000 g/mol (*n* ≈ 100,000 monomeric units). Bulk UHMW-PE exhibits a relatively low TC of 0.48 W/mK due to the random twisting and orientation of polymer chains, as well as the presence of numerous chain ends, vacancies, and defects that lead to high phonon scattering.^16,17^ There have been a number of strategies for the improvement of thermal properties of UHMW-PE. The key to obtaining intrinsically thermally conductive polymers is to structure the polymer chains into a regular arrangement in a particular direction, increasing the crystallinity. Engineering the polymer chain orientation to form ordered UHMW-PE fibers has shown improved mechanical strength and TC.^18^ A key factor affecting the TC of composites is the distribution of thermally conductive fillers in the UHMW-PE matrix.^19^ UHMW-PE doped with thermally conductive fillers has also attracted considerable attention such as boron nitride (BN),^20−22^ silicon nitride (Si_3_N_4_),^23^ aluminum nitride (AlN),^24^ aluminum oxide,^25^ silicon carbide (SiC),^26^ graphene,^27^ carbon nanotubes (CNTs),^28^ and nanomaterials. Furthermore, the incorporation of conductive BN via techniques like powder mixing and hot pressing has shown potential to further enhance TC. For instance, the TC of BN UHMW-PE composites with 38.3 vol % BN increased to 3.37 W/mK, seven times higher than that of bulk UHMW-PE. Nevertheless, the improvement in TC of polymer composites is constrained due to significant thermal interface resistance between the filler and the polymer matrix. These materials can also be configured into desired geometries along with layer structures to further optimize the performance for laser-induced damage. However, the solid-state extrusion technique has been demonstrated to significantly increase TC, reaching values as high as 104 W/mK, surpassing that of metals such as nickel, iron, and platinum.^15^ Consequently, UHMW-PE structures present promising alternatives for laser shielding applications.

We initially investigated the laser shielding properties of a 77-layered UHMW-PE ballistic material under 915 nm CW laser exposure at limited power levels (i.e., 100 W).^7^ In this study, we aim to explore the underlying physical mechanisms of laser-material interaction through multiphysics modeling and experimental validation for such layered UHMW-PE ballistic materials under high-power CW laser exposure (i.e., 5 kW at 1075 nm). To the best of our knowledge, this is the first time that both numerical modeling and experimental investigations have been conducted to assess and analyze the laser-induced damage mechanisms in layered UHMW-PE material structures in detail, with laser power extended up to 5 kW. Our laser-material interaction model considers the optical, thermal, and structural properties of these layered UHMW-PE composite materials. The numerical calculations from our model consistently align with experimental results, providing validated analysis of the laser-induced damage mechanisms concerning layered structure geometries and optical power and density (i.e., up to 5 kW at 1075 nm wavelength). Additionally, our findings reveal nonlinear behavior in certain cases.

## 2 Computational Methods

Spectra Shield is a trade name of UHMW-PE fibers which are the strongest and lightest fibers available and find a wide range of applications such as ballistic protection, medical, cut-resistant textiles, aviation, and in a variety of composite materials.^29^ Spectra Shield II grade SR-3136, manufactured by Honeywell using Spectra 3000 fiber, is commercially available in a continuous roll of thick fabric. This fabric is formed by pressing together four cross-laminated plates (0°/90°/0°/90°) into a single laminated layer. A piece of a roll of fabric can be cut to the desired dimension and shape to be molded in a hot press. Because polyethylene is a thermoplastic, it is used to fuse the fibers together by means of surface melting and recrystallization during applied heat and pressure. The production process begins by rolling out to cut the desired shape and putting each on top of each other in a crossed manner. After being consolidated into flat panels, the stacked layers are loaded into hot plates heated at 120–130 °C for pressing about 30 min under the 200 bar pressure. At this point, it is important that stacked layers must be evenly heated.^7^ Some of the physical properties of SR-3136 are outlined in Table 1.

**Table 1:** Physical Properties of Spectra Shield II Grade SR-3136

property name	Spectra Shield II grade SR-3136
manufacturer	Honeywell
density	0.97 g/cm^3^
fiber type	Spectra 3000
matrix resin type	polyurethane-based
resin weight	21 ± 3%
total weight	260 g/m^2^
elongation	3.75%
fiber diameter	29 μm
single laminated layer thickness (plies of 0°/90°/0°/90°)	400 μm
ply thickness	100 μm
avg. number of stacked fibers per ply thickness	2.7
melting point	150 °C

For the experiment, 10 cm × 10 cm UHMW-PE ballistic composite sheets were used. These sheets have a thickness of 400 μm for a single layer (0°/90°/0°/90°), a density of 0.97 g/cm^3^, and an in-plane TC of 20 W/mK. The samples used in the experiment were divided into four groups based on the number of layers: 1, 5, 10, and 15 layers.

During the experiment, three different near-infrared CW ytterbium fiber laser sources were employed. The first laser source was a tunable low-power laser (<25 mW), utilized for measuring the samples’ optical characteristics under room temperature conditions. The second (IPG-YLR-100-WC) and third (IPG-YLS-5000-SM) near-infrared laser sources were at a wavelength of 1075 nm with output power ranging from 100 W to 5 kW. The beam quality factor (*M*^2^) of these laser sources was less than 1.2, indicating single-mode laser output with a Gaussian beam profile. Water-cooled collimators were employed to collimate the beam after fiber output, with collimated beam diameters at the 1/*e*^2^ intensity level measuring 6.8 and 5 mm, respectively.

Throughout the experimental phase, two long-wave infrared (LWIR, 8–14 μm) thermal cameras were utilized to measure the temperature on the samples. The LWIR thermal cameras performed calculations based on the emissivity value of the object, humidity level, atmospheric transmissivity, distance of the object from the camera, temperature reflected from the surroundings, and surrounding temperature. Initially, both thermal cameras were set with identical parameters, with temperature ranges set from −20 to 650 °C. The recorded emissivity value was 0.95, with a reflected temperature of 20.0 °C, an atmospheric temperature of 20.0 °C, and a humidity level of 50%. These values were derived from the control unit of an ISO 8 class (10,000 class) clean room, where the experiments were conducted. The recording rate of the thermal camera was set to 30 Hz.

In this study, a 13 in. reflection and transmission integrating sphere (RTC), coated with a special material exhibiting a reflection value of 0.993 at a wavelength of 1075 nm in the inner region, was employed to measure the optical characteristics of the samples. The RTC sphere geometry facilitated both total and diffuse reflectance measurements. Utilizing the RTC sphere, the total reflectance and transmittance rate of samples were measured. Then, the absorbance of the samples was calculated by measuring reflectance and transmittance data at 1075 nm at room temperature using the following relation (eq 1):
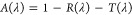
1where *A* represents the absorbance, *R* denotes the reflectance, and *T* is the transmittance of the sample at the laser wavelength.

Two-stage laser interaction experiments were conducted. In the first stage, the total (diffuse + specular) reflection and transmission values of four groups of UHMW-PE ballistic composite sheet samples were measured and recorded for each material. In this stage, the RTC sphere and a laser source with a maximum power of 25 mW and a wavelength of 1075 nm were utilized. In the second stage, the LIDT of each sample was investigated by varying the average laser output power, beam spot size, and operation duration. Laser power was adjusted from 11 to 100 W by utilizing the second laser source and from 250 W to 5 kW by using the third laser source, and the application duration ranged from 2 to 210 s. The output laser beam was collimated with spot sizes of 12 and 10 mm for the laser sources of 100 W and 5 kW, respectively. Additionally, throughout the 100 W laser, single-layered sample interaction experiments, the laser beam was also focused onto the sample surface with spot sizes of 4 and 2 mm, in order to increase laser beam fluency. Moreover, surface temperatures of the samples’ front and back surfaces were measured by using LWIR thermal cameras. While the experiments with a 100 W laser source were conducted in the ISO 8 class clean room, those conducted using a 5 kW laser source were carried out in a controlled outdoor high-power laser testing facility at the TÜBİTAK BİLGEM.

The interaction of 10 cm × 10 cm UHMW-PE composite samples with a high-power laser in the experiments is simulated via finite element analysis. The thermal behavior of the composite samples exposed to the laser beam for up to 210 s was studied by carrying out a time-dependent analysis. Inside the domain, the heat balance equation describing the temperature *T* (K) distribution due to laser heating is solved:
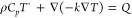
2Here, ρ represents density (kg/m^3^), *C*_*p*_ denotes heat capacity (J/(kg·K)), *k* stands for thermal conductivity (W/(mK)), and *Q* (W) represents the volumetric heat source. The laser irradiance of the beam is incorporated into the equation above through the volumetric heat source *Q*, which follows a Gaussian distribution on the in-plane axis centered at (*a*, *b*), and the Beer–Lambert law is applied along the optical axis (*z*):
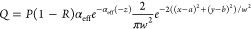
3where *P* represents the power (W) and *w* denotes the spot radius (m) of the laser beam measured on the sample surface (1/*e*^2^ half-width), and *R* and α_eff_ are the measured apparent reflectance (%) and calculated effective absorption coefficient (m^–1^) of the sample, respectively.

A NIR laser beam partially transmits through the UHMW-PE composite samples, resulting in multiple reflections within the materials. Consequently, the effective absorption coefficient can be deduced from the apparent transmittance *T* after reducing the percentage for the apparent reflectance. It can be calculated from the measured optical parameters (*R*, *T*) for each sample with a thickness *l* (m) as follows:
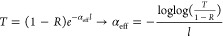
4

The phase change of the material is included in the model through an apparent heat capacity formulation. This method involves modifying the heat capacity using a smoothed function α_m_, which represents the fraction of phase before and after transition within a specified temperature interval with effective material properties:
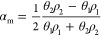
5where θ_1_ and θ_2_ are the volume fractions and ρ_1_ and ρ_2_ are the densities of the material before and after transition, respectively. These fractions satisfy the condition θ_1_ + θ_2_ = 1.

Subsequently, an equivalent heat capacity *C*_ep_ is introduced into the numerical model for the material with latent heat LH (J/kg) as follows:
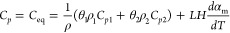
6

Additionally, the equivalent conductivity and density reduce to
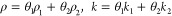
7

A ballistic composite structure with multiple layers of sheets consists of UHMW-PE fibers (79%) and polyurethane-based thermoplastic (21%), where a single UHMW-PE sheet consists of four layers of fibers oriented in 0°/90°/0°/90°. The anisotropic nature of the material properties is incorporated into the finite element model by calculating the effective material properties, which are presented in Table 2.

**Table 2:** Effective Material Properties of the Composite Samples Utilized in the Numerical Models^30^

	thermal conductivity along layers *k*_∥_ (W/(m·K))	thermal conductivity normal to layers *k*_⊥_ (W/(m·K))	density ρ (kg/m^3^)	heat capacity *C*_*p*_ (J/(kg·K))
UHMW-PE fibers	20.0	0.2	970	1850
polyurethane-based thermoplastic	0.2	0.2	970	1450–1700 @20 °C
				1700–1900 @80 °C
composite material effective	8.02	0.2	970	1766 @20 °C
				1819 @80 °C

Finally, convective and radiative heat flux boundary conditions are applied on the outer surfaces of the samples:
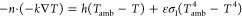
8where *T*_amb_ represents the ambient temperature, *h* is the heat transfer coefficient, ε is the emissivity of the surface, and σ_1_ is the Stefan–Boltzmann constant. The plate average heat transfer coefficient correlation with the laminar flow assumption is applied to simulate the cooling due to convection of air.^31^
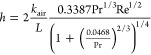
9where
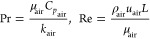
10

In these equations, μ_air_, *u*_air_, *Pr*, and *Re* represent the dynamic viscosity (Pa·s), velocity (m/s), and Prandtl and Reynolds numbers of air, respectively, all evaluated at . *L* is the characteristic length of the sample.

In the laboratory experiments, the air was almost still with a velocity of approximately 0.2 m/s, resulting in a heat transfer coefficient of approximately 5 W/m^2^K. In contrast, the outdoor experiments had an air velocity of approximately 2.8 m/s, leading to a heat transfer coefficient of around 20 W/m^2^K. Despite the increased velocity, the Reynolds number (1.7 × 10^4^) indicated that the flow remained within the limits of laminar flow (<5 × 10^5^).

## 3 Results and Discussion

In the experiments, the optical properties of UHMW-PE ballistic composite sheet samples were initially evaluated in terms of transmission, reflection, and absorption properties using an RTC sphere. Subsequently, high-power laser-induced ablation experiments in both laboratory and open-field settings were conducted to ascertain the LIDT values of the samples. The UHMW-PE ballistic composite sheets were methodically classified into four distinct groups based on their layer counts. Table 3 presents the average reflectance, transmittance, and absorbance values measured for the UHMW-PE ballistic composite sheet samples. Notably, as the number of layers increased, signifying the compression and formation of composite structures with varying layer counts, the absorbance of the samples exhibited a corresponding increase with a notable decrease in their transmittance. Interestingly, the reflectivity of the samples demonstrated a decrease up to five-layered samples, followed by an incremental rise in samples exceeding 5 layers. The variation in reflection and transmission among the different layered samples may arise from inherent surface diffusivity and alignment irregularities within the fibers composing the UHMW-PE ballistic sheets. Consequently, differing optical characteristics are observed across various regions of the samples.

**Table 3:** Measured and Calculated Optical Parameters of UHMW-PE Composite Samples

number of layers	number of samples	*R* (%)	*T* (%)		*A* = 1 – (*R* + *T*) (%)	
		measured data	measured data	utilized in numerical models	measured data	utilized in numerical models
1	1	66	38	34	0	0.4–0.6
5	2	62	36	36	2.2	1.7–2.2
10	3	65–70	25–28	25–31	4–7	3–7
15	2	69–71	17–19	18–22	8–11	5–9

In addition, 3D time-dependent finite element thermal simulations were conducted using COMSOL Multiphysics for the experiments to analyze the interaction between UHMW-PE composites and high-power laser beams. In these models, effective absorption coefficients are derived from measured reflectance and transmittance values. However, these values vary from sample to sample and even within the same sample across different regions. Additionally, the integrating sphere’s accuracy rate is approximately 3%. Consequently, while measured reflectance values are accepted without alteration in numerical models, parametric studies were conducted with transmittance values within a range of ±3% around the measured value for each sample. Maximum temperature values over time, obtained in the numerical calculations for each transmittance value, were compared with the corresponding experimental data. This iterative approach involves running simulations with varying transmittance values and selecting the one that closely matches the experimental temperature. Taking into account the measurement error and an additional variation of around 3% across samples and regions, this method allows for the derivation of the sample’s transmittance value through numerical analysis by accurately matching the experimental and calculated temperatures.

**Figure 1:**
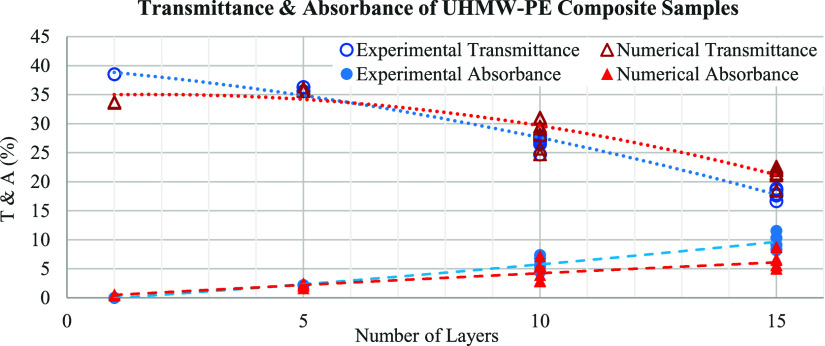
Experimental vs calculated transmittance *T* (%) and absorbance *A* (%) of UHMW-PE composite samples.

This phenomenon is particularly notable in the single-layered sample, where the absorbance of the composite is observed to be negligible (<0%) within the measurement certainties (i.e., mW), despite the sample heating up during the experiments. However, through parametric numerical analysis, this value is calculated to be between 0.4 and 0.6% (Table 3). Thus, the optimization technique employed aids in determining the correct transmittance and, subsequently, the absorbance of the corresponding sample. The calculated values of transmittance and absorbance obtained through numerical analyses are illustrated in Table 3 and Figure 1 alongside the experimental values for comparison.

### 3.1 UHMW-PE Composites Interacting with a Laser of Power up to 100 W

**Figure 2:**
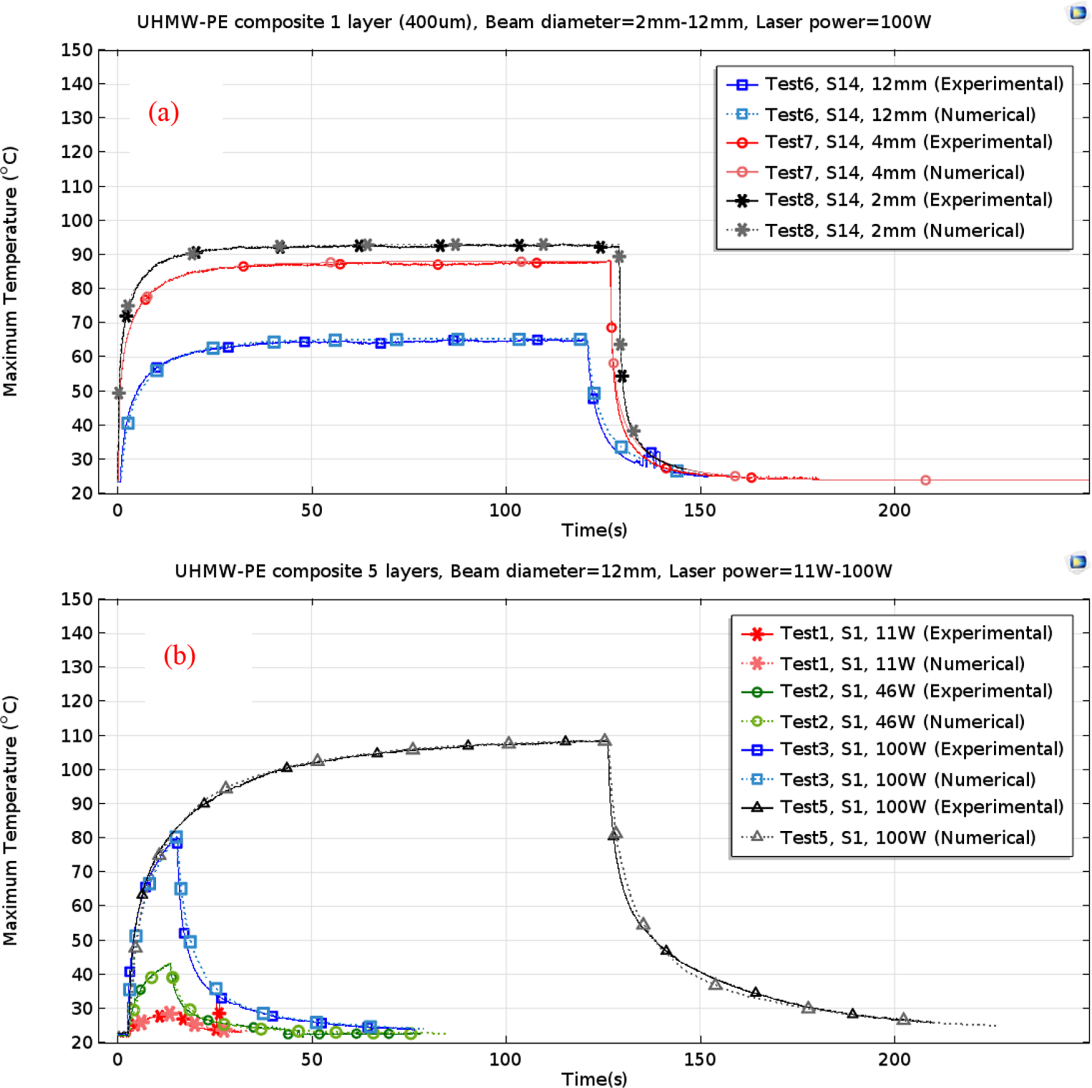
Comparison of experimental and numerical analyses of the temperature change induced by a laser on (a) single-layered and (b) 5-layered UHMW-PE samples: laser power is up to 100 W, and beam sizes vary at 2, 4, and 12 mm.

**Figure 3:**
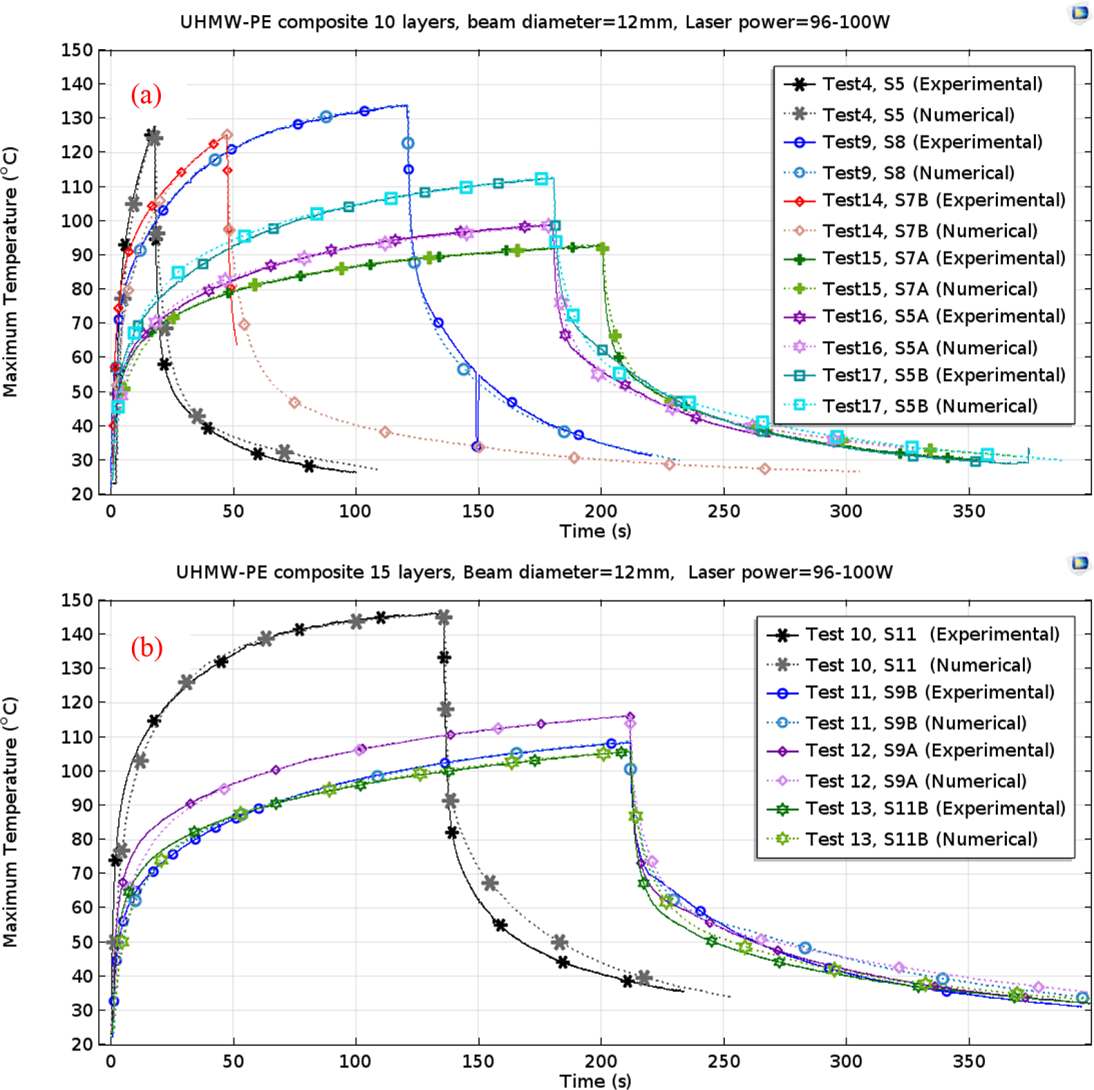
Comparison of experimental and numerical analyses of the temperature change induced by a laser on (a) 10-layered and (b) 15-layered UHMW-PE samples: laser power is approximately 100 W, and the beam size is 12 mm.

In determining the transient behavior of laser-induced temperature variations, samples were exposed to laser power up to 100 W for a maximum duration of 3 min in the experiments. The beam size on the targets was mostly 12 mm, but 2 and 4 mm beam sizes were also applied by focusing the collimated laser beams with an F150 lens. The maximum temperature of each sample was measured over time using a thermal camera. Numerically calculated maximum values for 1-, 5-, 10-, and 15-layered samples were compared with the measured data and are presented in Figures 2 and 3.

As the melting point of the composites is approximately 150 °C,^32^ none of the samples except one 15-layered sample (S11) were damaged during these tests (i.e., surface of the sample was slightly deformed).

### 3.2 UHMW-PE Composites Interacting with a Laser of Power above 100 W

Upon >100 W laser irradiation, samples exhibited distinct responses, particularly within the ablation zone, where notable transformations occurred. Here, the intense heat generated by the laser beam led to the melting of the material, followed by subsequent resolidification around the ablation zone. This phenomenon resulted in the formation of a discernible heat-affected zone (HAZ) surrounding the ablation area. Notably, the diameter of the ablation zone closely corresponds to the radius of the laser beam, indicative of the localized heating effect induced by the laser. However, the extent of the HAZ exceeded the dimensions of the beam spot, owing to the significant thermal expansion experienced by the sample upon exposure to the high-power laser. Furthermore, the vertical orientation of the sample during laser irradiation facilitated the downward redeposition of molten material, particularly evident in the lower portion of the HAZ zone. This observation underscores the complex interplay between laser-material interactions, ablation zone formation, and the thermal effects induced by high-power laser irradiation, all of which are crucial considerations in understanding the behavior of materials under such conditions.

**Figure 4:**
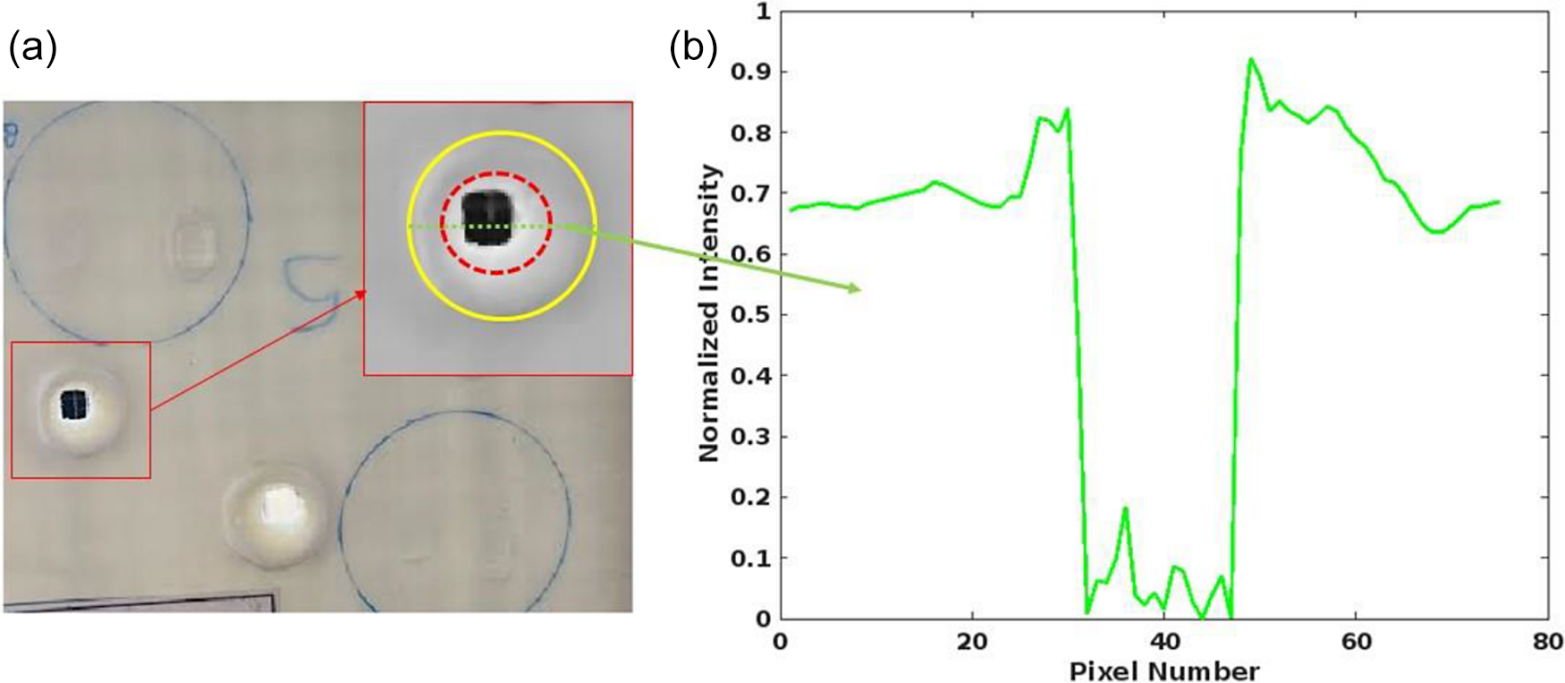
(a) UHMW-PE ballistic composite sheet after the laser ablation process. The inner dashed ring (red) defines the ablation zone, and the outer ring (yellow) indicates the heat-affected area. (b) Normalized intensity profile of the green line through the ablation area and HAZ with respect to its pixel line.

Figure 4 presents the typical surface morphology of the UHMW-PE ballistic composite sheet after exposure to a high-power CW near-infrared laser. Additionally, Figure 4b illustrates the intensity profile of the laser-induced damaged area. The intensity profile along a horizontal line through the affected area reveals the presence of shoulders in the HAZ near the ablation zone.

The investigation extended to analyzing the laser interaction zones of UHMW-PE ballistic composite layers under the influence of high-power near-infrared CW lasers, particularly focusing on surface temperatures. Figure 2 presents a graphical representation of the peak surface temperatures observed in single-layered, 5-layered, 10-layered, and 15-layered samples across different levels of laser fluencies. Notably, if the recorded maximum temperature of the back surface surpasses the 160–165 °C threshold, it signifies the commencement of complete moltenization within the ablated region. This observation is consistent with the melting temperature range documented in the existing literature,^32^ corroborating the findings of our study.

**Figure 5:**
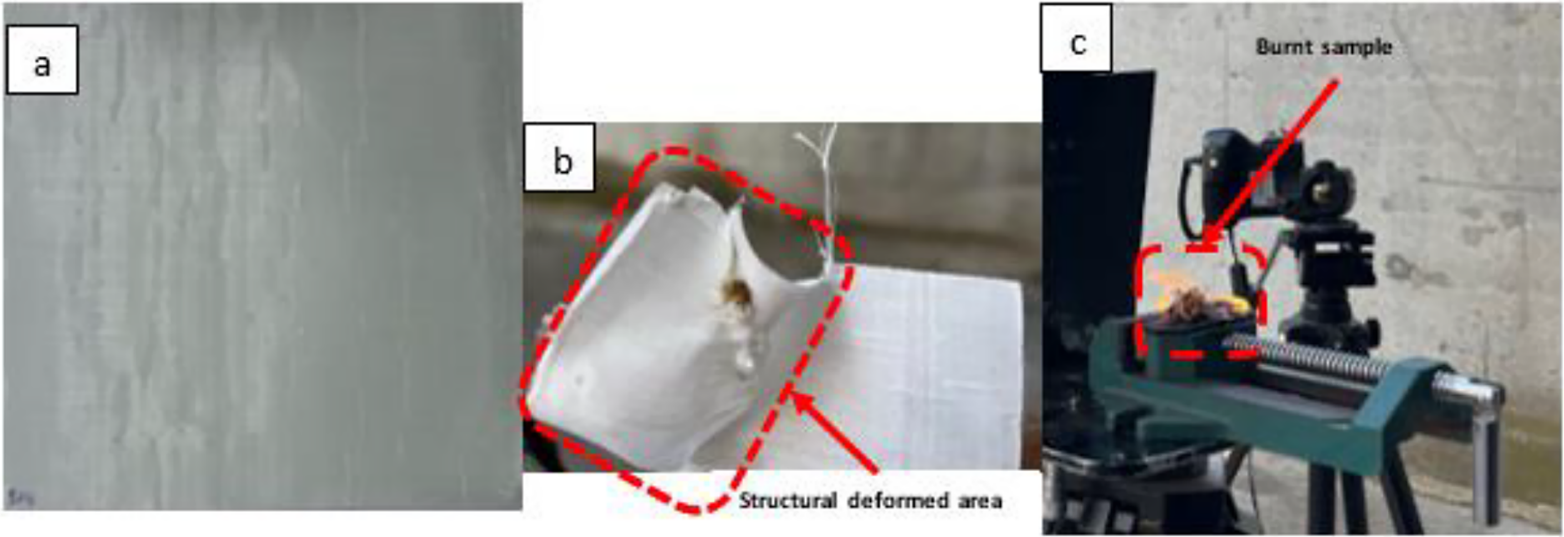
Three surface images of single-layered UHMW-PE ballistic composite sheets after near-infrared high-power CW laser irradiation. (a) Laser output power: 100 W, beam spot size: 1.8 mm, interaction duration: 120 s. (b) Laser output power: 850 W, beam spot size: 12 mm, interaction duration: 6 s. (c) Laser output power: 5 kW, beam spot size: 12 mm, interaction duration: 2 s.

For the single-layered samples, even when utilizing a 100 W laser source at maximum output power and focusing the beam onto the sample surface, which corresponds to a high level of laser fluency (approximately 5200 J/cm^2^), the sample’s temperature only reached 94 °C. Consequently, no observable morphological changes were noted on the surface of the single-layered sample as shown in Figure 5a. Subsequently, a new single-layered sample was subjected to irradiation by an 850 W laser output power with a 10 mm Gaussian beam spot size. As a result of the irregular thermal expansion of the ablated zone, the structure of the single-layered sample underwent complete deformation after 6 s of illumination, as shown in Figure 5b. Lastly, by further increasing the laser beam output power to the maximum level of 5 kW, the single-layered UHMW-PE ballistic composite sheet was completely damaged due to the laser-induced thermal load, as shown in Figure 5c.

**Figure 6:**
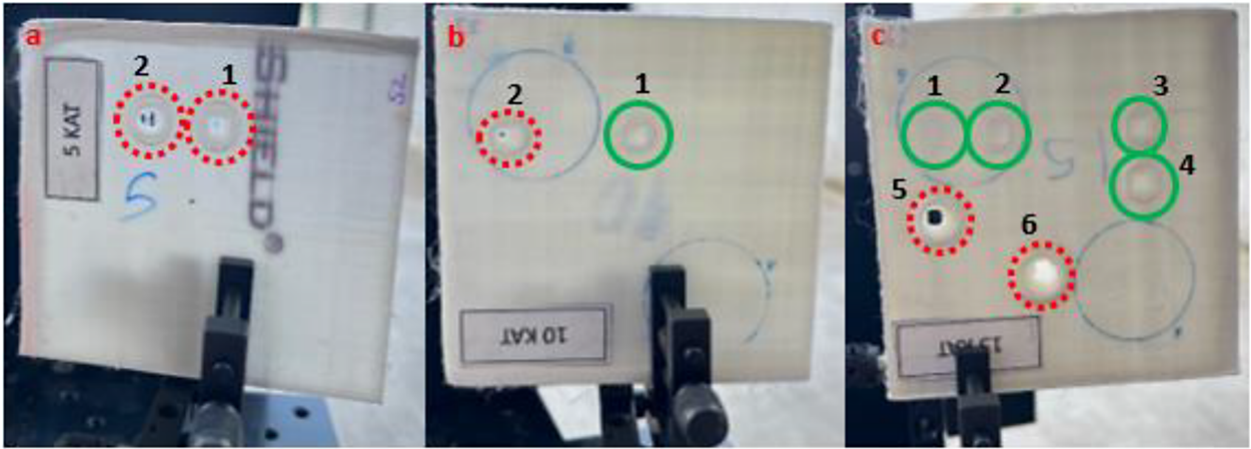
Four surface images of UHMW-PE ballistic composite sheets after near-infrared high-power CW laser irradiation with a 5 kW maximum output power. Dashed line circles (red) demonstrate the damaged region that has a hole; whole circles (green) indicate the region in which deformation started but no puncture occurred. (a) Sample with 5 layers, (b) sample with 10 layers, and (c) sample with 15 layers.

A comparative analysis of surface morphology among samples with varying layer numbers is presented in Figure 6, following irradiation by a high-power CW laser source with a collimated beam spot size of 10 mm. For the five-layered sample as shown in Figure 4a, two experiments were conducted utilizing laser output powers of 250 and 300 W as shown in Figure 6a1,a2, respectively. Here, the interaction durations are 30 and 19 s, respectively. In both cases, the measured maximum back surface temperature exceeded 165 °C, leading to complete melting of the ablation area. Subsequently, the 10-layered sample was exposed to a near-infrared laser beam with output powers of 290 and 300 W as shown in Figure 6b1,b2, for durations of 31 and 30 s, respectively. In the former scenario, deformation occurred without the formation of a hole. However, in the latter case with a 300 W laser beam, the maximum back surface temperature surpassed 165 °C, resulting in the formation of a hole in the ablation zone. Finally, the 15-layered UHMW-PE ballistic composite sample was subjected to irradiation by a near-infrared laser source with varying laser output powers: 200, 250, 260, 300, 350, and 5 kW for 30 s each as shown in Figure 6c1, c2, c3, c4, c5, and c6, respectively. Notably, perforation was observed only in the ablation area during the last two tests, wherein the maximum temperature reached on the back surface was approximately 175 °C.

**Figure 7:**
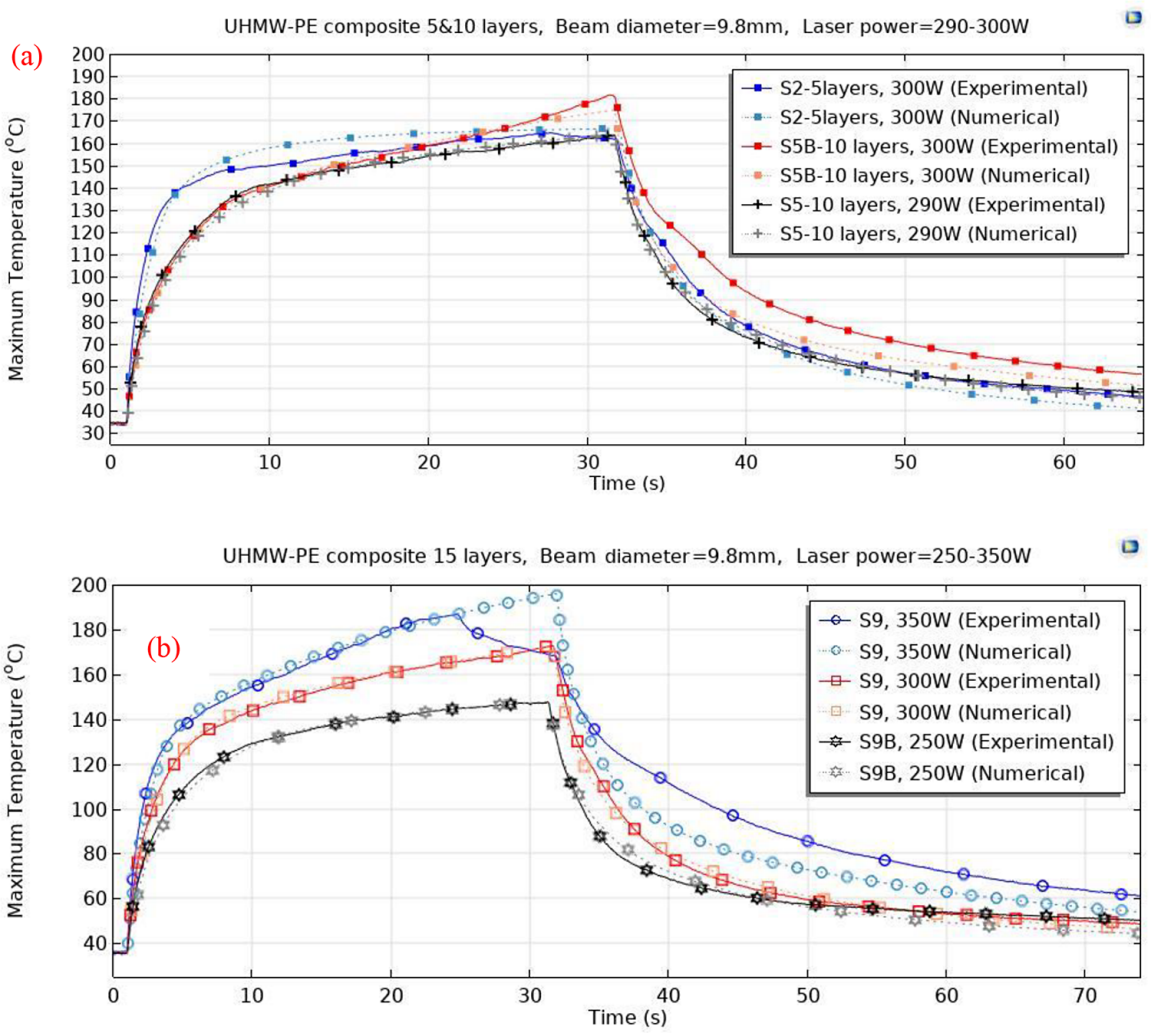
Comparison of experimental and numerical analyses of the temperature changes induced by a laser on (a) 5- and 10- and (b) 15-layered UHMW-PE samples: laser power varies from 250 to 350 W, and the beam size is 9.8 mm.

The thermal behavior during the experiments was further analyzed through simulations using the finite element method. The computed maximum temperatures for the specimens were subsequently compared with the empirically obtained data, as delineated in Figure 7. Despite the absence of consideration for topological alterations within these computational models, the peak temperatures observed on both the front and back surfaces closely mirrored those ascertained via thermal imaging until the emergence of a hole. This discrepancy between the front maximum temperatures after the perforation formation is notably conspicuous in the 15-layered specimen (S9), subjected to a 300 W laser power, where complete melting, indicated by the formation of a hole, occurred at approximately 25 s (Figure 7b) while laser exposure continued until 30 s. Subsequent to the formation of the perforation, a decrease in maximum temperature was observed on the front surface of the specimen, compared with a continued rise in the numerical simulations. To address the limitations posed by unaccounted topology changes, methodologies such as deformed geometry or moving mesh techniques could be applied in these numerical models. Nevertheless, given our primary focus on predicting the time of complete melting based on maximum temperatures, the utilization of numerical models disregarding topology changes suffices for investigating the phenomenon of damage in these materials.

### 3.3 LIDT and PIB Values for UHMW-PE Composites

LIDT values for the samples are calculated as follows:
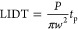
11where *w* is the beam spot radius of the laser applied on the sample (which is the same for all samples), *P* is the minimum power required for the formation of a perforation within 30 s, and *t*_p_ is the minimum time required for the formation of a perforation.

**Figure 8:**
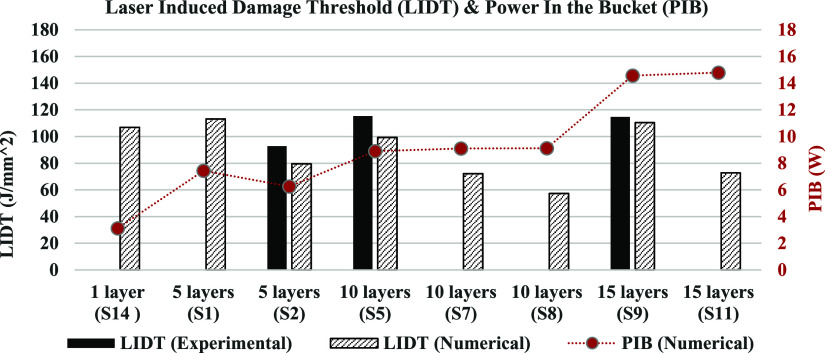
Numerically calculated and measured LIDT values of UHMW-PE ballistic composite samples with respect to their number of layers and PIB values.

Numerically calculated LIDT values for samples with 1, 5, 10, and 15 layers are presented alongside the measured data in Figure 8. Due to variations in the optical properties of the composite material among samples and within regions of a sample, the LIDT values do not exhibit a linear relationship with the number of layers. Based on the combination of numerical and experimental data, we assert that LIDT values range between 60 and 115 J/mm^2^ for the samples having 1–15 layers.

On the other hand, since the beam spot size is the same for all cases and the duration is held around 30 s for calculating the minimum power required for damage in almost all cases, the time and area terms in LIDT can be omitted. This simplifies the LIDT to units of power (W) instead of energy (J/mm^2^). Furthermore, as the material partially transmits the laser beam, only the portion of the power causing heating and subsequent damage may be considered in the threshold calculations. In other words, the total power absorbed by these samples, which transmit parts of the laser beam, can be conceptualized as “power in the bucket (PIB)” in a volumetric context:
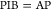
12where *A* represents the absorbance percentage of the sample with *P* denoting the minimum power required for the formation of a perforation within 30 s, akin to the concept described in LIDT.

Upon consideration of this metric for each case, a consistent relationship emerges among samples with the same number of layers, displaying a monotonically increasing trend with respect to the number of layers, as depicted in Figure 8. This is because thicker samples or samples with more layers tend to absorb more laser energy due to the increased interaction path length, requiring more laser power to achieve damage. The minimum absorbed power threshold for damage formation begins at approximately 3 W for the single-layered sample, increases with the number of layers, and reaches approximately 15 W for 15-layered samples. This increase is nearly linear for multiple-layered samples (≥5 layers). Calculations suggest that the absorbed power required for damage formation per layer ranges from approximately 1.0 to 1.4 W for samples with multiple layers.

PIB quantifies the absorbed laser power crucial for initiating damage to a material. It accounts for the absorbance of the sample and provides insights into laser-material interactions by focusing on effective heating within the required time period and damage-inducing power for a constant laser beam size. Understanding PIB helps optimize laser processing parameters and assess the material response so that one can predict and analyze how different materials or material configurations (such as layers in composite materials) will behave under specific laser conditions.

For the Spectra Shield studied in this case, a composite material with multiple layers, the PIB calculated from samples up to 15 layers can be used to estimate the damage threshold for any number of layers. For materials with more than 15 layers, the absorbance percentage can be determined either through measurement with a tool such as an integrating sphere or estimated by extrapolating data measured for up to 15 layers. Once the absorption is known, the power required for damage formation can be determined by using the PIB value calculated through numerical analysis.

It is worth noting that while absorbance tends to increase with the number of material layers, the reflectance values of the samples also correlate positively with the layer count. Furthermore, single-layered samples exhibit high laser beam transmission, but as they are compressed to form multilayered samples, internal reflections increase, thereby altering the laser’s propagation path. This phenomenon renders penetration increasingly challenging as the number of layers increases.

As a result, two figures of merit are used to understand and quantify the overall effect of the laser-matter interactions with UHMW-PE. First, LIDT defines the necessary power density to drill a hole in the material within a certain time interval. As the temperature increases due to laser irradiation, heat accumulates quickly, as observed through real-time temperature monitoring. Due to its relatively low TC, which lowers the LIDT, the material undergoes a phase transition (melting), which is included in the model through its heat capacity. Second, although the UHMW-PE composite is partially transparent to the laser, the presence of reinforcing fibers and microstructural changes, such as defects, impurities, and voids, can scatter and absorb more light, eventually reducing the overall transmission. Therefore, absorbance plays a critical role in damaging the target area with minimal power within a given time. Therefore, PIB is a critical parameter, representing the effective delivery of laser power to a target area. It is both experimentally and numerically proven that this study reveals the nonlinear behavior of UHMW-PE against laser heating with increasing layers as can be inferred from Table 3.

Although the mechanical durability of the material as an armor is well-known and proven against projectiles and metal fragments, it becomes much less durable against laser beams, especially at high fluency. Based on our work, we propose some suggestions to manage heat dissipation. Incorporating higher TC materials such as metals, metallic glasses, or carbon-based materials within the layered structure of UHMW-PE to exhibit composite hybrid behavior can help improve heat dissipation. Surface coatings or treatments that reflect or absorb laser energy more effectively or enable lateral spreading of heat caused by laser irradiation can help reduce the thermal load on the UHMW-PE layers. It is evident that there is a need for further research to reinforce the UHMW-PE composite with secondary materials and/or nanoparticles to be used as a hardener against laser beams without degrading its mechanical strength.

## 4 Conclusions

The study successfully validated a 3D time-dependent finite element numerical model for analyzing laser-induced damage in UHMW-PE composites with varying layer configurations. To the best of our knowledge, this is the first time that both numerical modeling and experimental investigations have been conducted to assess and analyze the laser-induced damage mechanisms in layered UHMW-PE material structures in detail, with laser power extended up to 5 kW. The numerical results closely aligned with experimental data, demonstrating that laser-induced damage increases with the number of layers due to higher absorption and internal reflections. The experiments revealed that the minimum absorbed power threshold for damage initiation is approximately 3 W, increasing to around 15 W for the 15-layered samples. LIDT values for the samples ranged between 60 and 115 J/mm^2^, highlighting the nonlinear behavior in transmission, absorption, and scattering with an increasing number of layers.

These findings provide critical insights for the development of more laser-resistant materials and structures. For practical shielding applications, it is essential to limit transmission by enhancing absorption or scattering of laser illumination and ensuring high thermal durability with improved TC and transition temperature. The combination of numerical and experimental data underscores the importance of optimizing the internal structure of UHMW-PE composites to achieve higher resistance to laser-induced damage.

## Data Availability

The data that support the findings of this study are available from the corresponding author (E.O.) upon reasonable request as it is unpublished data for another study.
